# Single-cell mass cytometric analysis of peripheral immunity and multiplex plasma marker profiling of non-small cell lung cancer patients receiving PD-1 targeting immune checkpoint inhibitors in comparison with platinum-based chemotherapy

**DOI:** 10.3389/fimmu.2023.1243233

**Published:** 2023-10-13

**Authors:** Patrícia Neuperger, Klára Szalontai, Nikolett Gémes, József Á. Balog, László Tiszlavicz, József Furák, György Lázár, László G. Puskás, Gábor J. Szebeni

**Affiliations:** ^1^ Laboratory of Functional Genomics, HUN-REN Biological Research Centre, Szeged, Hungary; ^2^ PhD School in Biology, University of Szeged, Szeged, Hungary; ^3^ Csongrád County Hospital of Chest Diseases, Deszk, Hungary; ^4^ Department of Pathology, University of Szeged, Szeged, Hungary; ^5^ Department of Surgery, University of Szeged, Szeged, Hungary; ^6^ Avicor Ltd., Szeged, Hungary; ^7^ Department of Physiology, Anatomy and Neuroscience, Faculty of Science and Informatics, University of Szeged, Szeged, Hungary; ^8^ CS-Smartlab Devices Ltd., Kozármisleny, Hungary

**Keywords:** non-small cell lung cancer, platinum-based chemotherapy, PD-1 blocking, Nivolumab, Pembrolizumab

## Abstract

**Introduction:**

The effect of platinum-based chemotherapy (Chem.) and second- or multiple- line immune checkpoint PD-1 blocking therapy by Nivolumab or Pembrolizumab (ICI) was assayed in the peripheral blood of non-small cell lung cancer (NSCLC) patients.

**Methods:**

Flow cytometry was used to detect NSCLC-related antigen binding IgG antibodies. The Luminex MagPix multiplex bead-based cytokine/chemokine detecting system was used to quantitatively measure 17 soluble markers in the plasma samples. Single-cell mass cytometry was applied for the immunophenotyping of peripheral leukocytes.

**Results:**

The incubation of patient derived plasma with human NSCLC tumor cell lines, such as A549, H1975, and H1650, detected NSCLC-specific antibodies reaching a maximum of up to 32% reactive IgG-positive NSCLC cells. The following markers were detected in significantly higher concentration in the plasma of Chem. group versus healthy non-smoker and smoker controls: BTLA, CD27, CD28, CD40, CD80, CD86, GITRL, ICOS, LAG-3, PD-1, PD-L1, and TLR-2. The following markers were detected in significantly higher concentration in the plasma of ICI group versus healthy non-smoker and smoker controls: CD27, CD28, CD40, GITRL, LAG-3, PD-1, PD-L1, and TLR-2. We showed the induction of CD69 and IL-2R on CD4+ CD25+ T-cells upon chemotherapy; the exhaustion of one CD8+ T-cell population was detected by the loss of CD127 and a decrease in CD27. CD19+CD20+, CD79B+, or activated B-cell subtypes showed CD69 increase and downregulation of BTLA, CD27, and IL-2R in NSCLC patients following chemotherapy or ICI.

**Discussion:**

Peripheral immunophenotype caused by chemotherapy or PD-1 blocking was shown in the context of advanced NSCLC.

## 1 Introduction

Lung cancer, the most common cancer type causes approximately 13% of all cancer deaths worldwide (1). Lung cancer is a heterogeneous disease classified by histology into two major types: small-cell lung carcinoma (22%) and non-small-cell lung carcinoma (NSCLC), which is further classified into adenocarcinoma (40%), squamous cell carcinoma (30%), and large cell carcinoma (8%) (2). The overall 5-year survival rate is approximately 15% for non-small cell lung cancer and approximately 6% for small-cell lung carcinoma (3, 4). Tobacco smoking has been described to be responsible for 87% of all lung- cancer-related deaths in the USA (5). Although both types are affected differently by tobacco smoking, it has been proven that tobacco smoke is the main preliminary environmental causative factor for lung cancer (2). The main therapeutic options are surgery, radiation therapy, chemotherapy, targeted therapy of the driver mutations of cancer cells, or immunotherapies. A combination of these therapies can be also used following recent guidelines and local recommendations. Platinum-based chemotherapeutics, such as cisplatin, carboplatin, and oxaliplatin, are gold standard chemotherapy treatment options for lung adenocarcinoma without targetable mutation (6, 7). In the advent of immunotherapy, the application of ICIs has dramatically changed patient overall survival (OS) of well-responders; first, PD-1 blocking Nivolumab and Pembrolizumab were applied as second-line treatment options showing superior objective response rate (ORR) and OS compared to docetaxel in NSCLC (8–10). Unfortunately, the tumor progression often outperforms initial response, or resistance to ICI also may develop (11). Several immune mechanisms may counteract with the success of ICI therapy, such as T-cell exhaustion, decreased antigen presentation, altered metabolism, or downregulation of co-stimulatory molecules (12). Although PD-L1 expression is a strong indication, we still lack prognostic markers that could increase patient benefit to PD- 1-targeting ICI therapy (13).

We focus here on the peripheral immune compartment in smoker lung adenocarcinoma patients receiving cisplatin/carboplatin chemotherapy or second- or multiple-line PD-1- targeting immunotherapy. It has been widely known that most of the malignancies bear tumor antigens; carcinogenic compounds of tobacco smoke *per se* generates mutations in the lung, making tissues more immunogenic. However, continuous tobacco smoking triggers a myriad of immune reactions; the activation state and polarization of both myeloid and lymphoid cells are affected in smoker lung cancer patients, making the immune infiltrate irresponsive, the so- called tolerogenic toward arising malignant cells (14). The emergence of myeloid-derived suppressor cells, M2 macrophages, and regulatory T-cells and production of transforming growth factor-β, IL-10, or PD-L1 may sustain the tumor- prone microenvironment (15–17). The deeper insight into the heterogeneity of inflammatory cells in the blood could help to understand the mechanisms responsible for the switch from a tumor suppressor to a tumor promoter immunophenotype. The knowledge of multi-cellular phenotypes and molecular mechanisms responsible behind chronic inflammation and tolerance toward malignancy in lung cancer could reveal novel therapeutic targets. The immune system, due to its high plasticity, can represent different polarization states. Upon activation, innate hematopoietic cells infiltrate the respiratory tract, generating pulmonary inflammation *via* TLR4/MyD88 and IL-1R1/MyD88 signaling- dependent mechanisms (18–20). Among others, we have previously reviewed how tobacco smoking may pave the way for chronic obstructive pulmonary disease (COPD) frequently leading to lung cancer (3, 21). Our interest turned toward the immunophenotyping of peripheral mononuclear cells (PBMCs) comparing smoker NSCLC cases who underwent first- line chemotherapy with cases receiving second/multiple line PD-1- blocking therapy. Our goal was to understand better the obstacles to boost antitumor immunity or overcome tumor- induced tolerance. We aimed to identify leukocyte subsets that are capable of suppressing tumor development; moreover, we aimed to identify leukocyte subsets that are immunosuppressive and unable to mount an effective anti-tumor immune response in lung cancer patients.

## 2 Materials and methods

### 2.1 Cell culturing

Cell culturing was performed as described previously by our group (22). Briefly, the human non-small cell lung cancer (NSCLC) cells, namely, adenocarcinoma cell lines A549, H1975, and H1650, were purchased from the American Type Culture Collection. The H1975 and H1650 cells were maintained in Dulbecco's Modified Eagle Medium (DMEM) or A549 cells in DMEM/F12 (DMEM, PAN-Biotech GMBH, Aidenbach, Germany; DMEM/F12 Nut mix, Gibco, Thermo Fisher Scientific, Waltham, MA, USA) containing 4.5 g/L glucose, 10% fetal bovine serum (FBS) (Gibco), 2 mM GlutaMAX (Gibco, Waltham, MA, USA), 100 U/ml penicillin, and 100 g/ml streptomycin antibiotics (penicillin G sodium salt and streptomycin sulfate salt, Sigma-Aldrich, St. Louis, MO, USA). The cells were cultured in a standard tissue culture Petri dish, 10 mm in diameter (Corning Life Sciences, Corning, NY, USA) at maximum 80% confluence in a standard atmosphere of 95% air and 5% CO_2_.

### 2.2 Study design

Subjects were recruited from the following groups: (1) non-smoker healthy control (without known disease and without regular medication, n=12), (2) smoker lung NSCLC patients receiving first-line chemotherapy (Chem., n=10, only one case was non-smoker), and (3) smoker lung adenocarcinoma receiving second- or multiple- line immunotherapy, where first- line chemotherapy was already terminated before starting the immunotherapy (ICI, n=10). Plasma samples of healthy smoker controls (n=9) were available for multiplex quantitative analysis of soluble markers using the Luminex MagPix system. These healthy smoker controls were recruited with minimum 5 years smoking history with minimum of 10 cigarettes per day without the awareness of chronic illness and without regular medication. The experimental procedures are cross-sectional with the collection and the analysis of 20 ml venous peripheral blood at one time point. However, the follow-up of the patients provided progression- free survival (PFS) and OS data (**Table 1**). The immunotherapy significantly improved the OS (**Supplementary Figure S1**). Patients were included with histologically or cytologically confirmed NSCLC (primarily squamous cell or adenocarcinoma) and patients with stage IV or selected stage IIIB disease by the International Staging System (lung cancer). Stage IIIB patients had to have a positive pleural effusion or multiple ipsilateral lung nodules (potentially inoperable disease). Inclusion criteria were bidimensional measurable or assessable disease, PS (performance status) of 0 or 1. Previous surgery and radiotherapy were allowed. One group of patients received first-line platinum-based chemotherapy treatment (Chem.), and the other group received second- or multiple-line immunotherapy after failed chemotherapy (ICI) (in accordance with the Hungarian financing protocol). The samples for the Chem. group were isolated following a minimum of four cycles of platinum-based chemotherapy. The samples for the ICI group were isolated when initial chemotherapy was already terminated, and the following ICI therapy was applied for at least 3 months.

**Table 1:** Demographic and clinical characteristics of the enrolled patients.

Subjects	Age (years) Median ± SD	Female	Lung cancer histology	Therapy See details in **Supplementary Table S1**	Progression- free survival to initial therapy (month) Median ± SD	Overall survival (month) Median ± SD
Control	60.5 ± 7.6	50%	none	none	Not relevant	Not relevant
Smoker Control	54 ± 7.3	66%	none	none	Not relevant	Not relevant
Chem.	67.3 ± 5.6	40%	50% adenocarcinoma 50% squamous cell c.	Cisplatin, or Carboplatin, or combined with Pemetrexed, or Gemcitabine	11 ± 13.4	14 ± 20.5 months
ICI	65.1 ± 5.0	50%	60% adenocarcinoma, 30% squamous cell, c. 10% adenocarcinoma + squamous cell c.	Second-line Nivolumab or Pembrolizumab, min. 6 cycles	11 ± 25.9	59.5 ± 45.8 months

Patient’s clinical data are summarized in **Supplementary Table S1**.

### 2.3 Ethical statement

The subjects gave their informed consent for inclusion before participating in the study. The study was conducted in accordance with the Declaration of Helsinki, and the protocol (“Immunophenotyping in COPD and lung cancer”) was approved by the Ethics Committee of the National Public Health Center, Hungary under the 33815-7/2018/EÜIG Project identification code and by the Ethics Committee of the University of Szeged under the 163/2018-SZTE Project identification code.

### 2.4 PBMC isolation

After the collection of 20 ml of blood into an EDTA vacutainer (Becton Dickinson, Franklin-Lakes, USA), PBMCs were purified by Leucosep tubes (Greiner Bio-One, Kremsmünster, Austria) according to the manufacturer’s instructions. Plasma samples were harvested, aliquoted, and stored at −80°C. If the pellet was light red, 2 mL ACK Lysing Buffer (ACK: 0.15 M NH_4_Cl, 10 mM KHCO_3_, 0.1 mM Na_2_EDTA, pH 7.3; Merck, Darmstadt, Germany) was applied at room temperature (RT) for 2 min. Samples were washed twice with 10 ml phosphate-buffered saline (PBS) (Merck), and subsequently, cell count and viability check were performed with Trypan Blue (Merck) exclusion. Cryopreservation of PBMCs was carried out in stocks of 4 × 10^6^ cells of 1 ml FBS (Capricorn Scientific, Ebsdorfergrund, Germany) supplemented with 1:10 dimethyl sulfoxide (DMSO) (Merk) [v/v] in cryotubes (Greiner Bio-One) in liquid nitrogen (Messer, Bad Soden, Germany).

### 2.5 Tumor- cell- specific antibody binding assay

The supernatant of A549, H1975, or H1650 cells grown in 80% confluence in 10 mm diameter Petri dish was removed, and cells were washed with 5 ml PBS and detached by 2 ml Accutase (Thermo Fisher Scientific), centrifuged at 350*g*, 5 min, washed by 5 ml PBS, centrifuged at 350*g*, 5 min, and resuspended in 1ml IFB (Immune Fluorescence Buffer: PBS with 2% FCS). Cells were counted using a Bürker chamber and Trypan Blue, and 2 × 10^5^ viable cells were pipetted into a 1. 5-ml tube (Eppendorf) in 50 µl IFB. Plasma samples were diluted 1:1 in IFB, 25µl + 25 µl, and added to the cells, incubated in 100 µl final volume (4× dilution of the plasma) at 4°C for 60 min. Samples were washed with 1 ml IFB, centrifuged 350*g*, 5 min. Secondary antibody anti-human Alexa488 (Cat. num. 409322, clone HP6017, BioLegend) detecting IgG antibodies (IgA and IgM is not detected) was added in 1:25 dilution in IFB for 30 min at 4°C. Cells were resuspended in 300 µl IFB; 10 µg/ml propidium iodide was added right before the acquisition by FACS Calibur (Becton Dickinson) to gate out dead cells. A schematic cartoon of the assay is illustrated in **Supplementary Figure S2**. Manual gating was used in CellQuest (Beckton Dickinson) analyzing PI-negative but anti-human-Alexa488- positive cells (**Supplementary Figure S3**).

### 2.6 Measurement of plasma proteins

The measurement of plasma proteins was performed as described previously by our group with minor modifications (23, 24). Briefly, after the withdrawal of 20 ml blood into an EDTA vacutainer (Becton Dickinson), human peripheral blood mononuclear cells and plasma samples were purified by Leucosep tubes (Greiner Bio- One). Plasma fractions were stored at −80°C in aliquots before running the assay. Luminex xMAP (MAGPIX^®^) technology was used to determine the protein concentrations of 17 distinct soluble mediators (BTLA, CD28, CD80, CD27, CD40, CD86, CTLA-4, GITR, GTRL, HVEM, ICOS, LAG-3, PD-1, PD-L1, PD-L2, TIM-3, and TLR-2) performing the Human Immuno-Oncology Checkpoint Protein Panel 1— Immuno-Oncology Multiplex Assay (Cat. num. HCKP1-11, Merck) according to the instructions of the manufacturer. Briefly, all samples were thawed and diluted with sterile PBS to 1:1 and were tested in a blind fashion and in duplicate. A total of 25 μl volume of each sample, standard, and universal assay buffer was added to a 96-well plate (provided with the kit) containing 50 μl of capture antibody-coated, fluorescent-coded beads. Biotinylated detection antibody mixture and streptavidin- PE were added to the plate after the appropriate incubation period. After the last washing step, 120 μl reading buffer was added to the wells, and the plate was incubated for an additional 5 min and read on the Luminex MAGPIX^®^ instrument. Luminex xPonent 4.2 software was used for data acquisition. Five-PL regression curves were generated to plot the standard curves for all analytes by the Analyst 5.1 (Merck) software, calculating with bead median fluorescence intensity values. The scatter plots of the individual values are demonstrated in **Supplementary Figure S4**. The panel of the investigated 17 plasma proteins and the range of the detection (in pg/ml from the lower limit to the upper limit) are available in **Supplementary Table S2**. Data were pooled from two independent measurements and plotted in GraphPad Prism v8 (Dotmatics, Boston, USA).

### 2.7 Cell preparation for CyTOF (cytometry by time-of-flight)

Cells were processed for CyTOF as described previously by our group (25). Briefly, cryotubes were thawed in a 37°C water bath, and cells were transferred into 14 ml of 37°C warm RPMI 10% FBS (Capricorn Scientific) and centrifugated at 350*g* for 6 min at RT. PBMCs were washed again with 10 ml RPMI 10% FBS, and cells were counted and viability determined with Trypan Blue exclusion. PBMCs, 2.5 × 10^6^ cells/sample, were plated on a 96-well repellent plate (Greiner Bio-One) separately in 200 µl RPMI 10% FBS. After 2 h, cells were stimulated with 10 ng/ml phorbol myristate acetate (PMA), 1 µg/ml ionomycin, and 5 µg/ml Brefeldin A for 16 h in an incubator with 5% CO_2_ at 37°C. Next, cells were collected and washed twice with Maxpar Cell Staining Buffer (MCSB; Fluidigm, South San Francisco, USA).

### 2.8 Antibody staining

The antibody staining of cells for CyTOF was performed as described previously by our group with minor modifications (26, 27). Before harvesting, cells were incubated with 100 mM EDTA for 15 min RT. Briefly, viability was determined by cisplatin (5 µM ^195^Pt, Fluidigm) staining for 3 min on ice in 500 µl PBS. The sample was diluted by 3 ml Maxpar Cell Staining Buffer (MCSB, Fluidigm) and centrifuged at 350 g for 5 min. Cells were resuspended in 50 µl MCSB supplemented with 1:20 v/v TrueStain FcX™ FC receptor blocking solution (Biolegend, San Diego, USA) and incubated at RT for 10 min. Without a washing step, samples were barcoded by adding 50 µl of different metal-tagged (A-^89^Y, B-^112^CD, C-^113^CD, D-^114^CD, and E-^116^CD) CD45 antibodies (clone: 30-F11; Fluidigm) at a final concentration of 1:100 v/v per antibody and incubated at 4°C for 30 min. The codes of the barcoding were the following: 1. AB, 2. AC, 3. AD, 4. AE, 5. DC, 6.BD, 7. BE, 8. CD, 9. CE, and 10. DE. Following the barcoding, 10 samples were pooled for the subsequent antibody staining. First, cells were stained with cell surface antibodies from our Immune Checkpoint Panel designed in-house and bought antibodies from Fluidigm and incubated at 4°C for 30 min, washed twice with 2 ml MCSB. The list of the antibodies used for the study is in **Table 2**. Fixation was performed with 1 ml Maxpar Fix I buffer (5×) diluted in PBS, incubated at RT for 30 min, and washed twice with 2 ml Maxpar PermS Buffer, centrifugated at 800*g*, 5 min. Cells were stained with the intracellular markers (TNF-α, IL-6, IL-2, granzyme B, and perforin) and incubated at RT for 30 min. Cells were washed twice with MCSB and fixed with 1 ml Pierce™ formaldehyde (Thermo Fisher Scientific) solution diluted in PBS to 1.6% and incubated at RT for 10 min. Stained and fixed cells were centrifuged at 800 g at RT for 6 min and resuspended in 800 µl Fix & Perm solution (Fluidigm) supplemented with 1:1,000 v/v ^191^Ir-^193^Ir DNA intercalator (Fluidigm) for overnight incubation.

**Table 2:** The list of the antibodies used for the mass cytometry.

Marker	Clone	Metal tag	Supplier
Gal-1	2C1/6	^141^Pr	Monostori’s lab (28)
CD40	5C3	^142^Nd	Fluidigm
CD5	SK1	^143^Nd	Fluidigm
CD69	FN50	^144^Nd	Fluidigm
CD138	DL-101	^145^Nd	Fluidigm
CD11c	3.9	^146^Nd	Fluidigm
CD20	2H7	^147^Sm	Fluidigm
IgA	Polyclonal	^148^Nd	Fluidigm
CD86	IT2.2	^150^Nd	Fluidigm
HLA-DR	G46-6	^151^Eu	Fluidigm
TNF-α	Mab11	^152^Sm	Fluidigm
Mac-2/Gal-3	M3/38	^153^Eu	Fluidigm
CD3	UCHT1	^154^Sm	Fluidigm
CD279 (PD-1)	EH12.2H7	^155^Gd	Fluidigm
IL-6	MQ2-13AS	^156^Gd	Fluidigm
CD134 (OX40)	ACT35	^158^Gd	Fluidigm
CD274 (PD-L1)	29E.2A3	^159^Tb	Fluidigm
CD28	CD28.2	^160^Gd	Fluidigm
CD80 (B7-1)	2D10.4	^161^DY	Fluidigm
CD79B	CB3-1	^162^Dy	Fluidigm
CD272 (BTLA)	NIH26	^163^Dy	Fluidigm
CD19	HIB19	^165^Ho	Fluidigm
IL-2	MQ1-17H12	^166^Er	Fluidigm
CD27	L-128	^167^Er	Fluidigm
CD8a	SK1	^168^Er	Fluidigm
CD25 (IL-2R)	2A3	^169^Tm	Fluidigm
CD152 (CTLA-4)	14D3	^170^Er	Fluidigm
Granzyme B	GB11	^171^Yb	Fluidigm
IgM	MHM-88	^172^Yb	Fluidigm
CD4	SK3	^174^Yb	Fluidigm
Perforin	B-D48	^175^Lu	Fluidigm
CD127	A019D5	^176^Yb	Fluidigm
CD16	3G8	^209^Bi	Fluidigm
CD45	HI30	^89^Y	Fluidigm
CD45	Hl30	^112^Cd	Fluidigm
CD45	Hl30	^113^Cd	Fluidigm
CD45	Hl30	^114^Cd	Fluidigm
CD45	Hl30	^116^Cd	Fluidigm

### 2.9 CyTOF data acquisition and data preprocessing

The acquisition of the samples for CyTOF was executed as described previously by our group with minor modifications (25, 27). Briefly, samples were washed twice with MCSB and once with PBS prior filtered through a 30-μm Celltrics (Sysmex, Bornbarch, Germany) gravity filter, and the cell concentration was adjusted to 7×10^5^/ml in Maxpar Cell Acquisition Solution (Fluidigm). Finally, EQ four- element calibration beads (Fluidigm) were added at a 1:10 ratio (v/v) and acquired on a properly tuned Helios mass cytometer (CyTOF, Fluidigm). We collected 1 × 10^6^ events per barcoded sample. The generated flow cytometry standard (FCS) files were randomized and normalized with the default setting of the internal FCS-processing unit of the CyTOF Software (Fluidgm, version:7.0.8493). The analysis was carried out in Cytobank (Beckman Coulter) by manual gating. The gating hierarchy is shown in **Supplementary Figure S5**. The raw median values were exported from Cytobank and archinh transformed in MS Excel.

### 2.10 Statistical analysis

Data were analyzed with GraphPad Prism 8.0.1. Normality of distributions was tested with D’Agostino and Pearson test with an 0.05 alpha value. We used non-parametric Kruskal–Wallis test for the four group comparisons with non-normal distribution. Dunn’s test was used for multiple comparisons. The log-rank test was used for OS data. Differences are considered significant at *p < 0.05, **p < 0.01, and ***p < 0.001.

## 3 Results

### 3.1 Platinum-based chemotherapy and second- or multiple-line PD-1 blockade of well-responders increased humoral immunity in NSCLC patients

**Figure 1:**
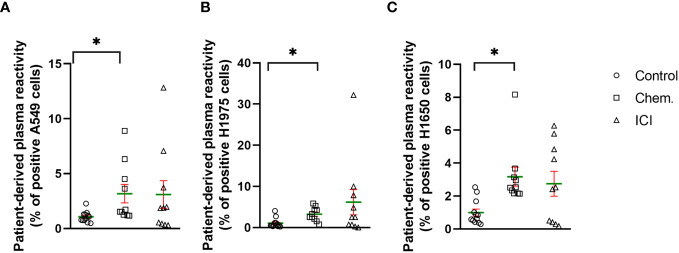
Platinum-based chemotherapy and PD-1 blockade increased the production of tumor cell surface-specific IgG antibodies in well-responder NSCLC patients. The scatter plots demonstrate the effect of platinum-based chemotherapy (Chem., n=10) or second- line immunotherapy (ICI, n=10) to the level of IgG antibodies in the plasma of patients with reactivity of **(A)** A549, **(B)** H1975, or **(C)** H1650 NSCLC cell line cell surface epitopes. Age- and gender-matched healthy controls were recruited without known illness (Control, n=12). The experimental details can be found in the *Materials and Methods.* Briefly, flow cytometry was used to detect human NSCLC cell- line-specific cell surface binding IgG antibodies. *p < 0.05.

Among others, we have previously shown the immunomodulatory effects of platinum-based chemotherapeutics, such as cisplatin or carboplatin used in the current study (25, 29, 30). Here, we developed a flow- cytometry-based assay to measure the increase in NSCLC-related cell surface epitope binding IgG antibodies in cancer patients. The incubation of patient-derived plasma with human NSCLC tumor cell lines, such as A549, H1975, and H1650, were used to detect NSCLC -specific antibodies. The percentage of reactive A549 cells in control samples were between min–max of 0.4% –2.2% (mean, 1; SD, 0.4; SEM, 0.1) compared to the chemotherapy- treated group reaching a maximum of 8.9% of cells (min–max, 1.1% –8.9%; mean, 3.1; SD, 2.6; SEM, 0.8; *p<0.05). The ICI treatment led to the production of A549 binding antibodies between min–max of 0.3%–12.8% (mean, 3; SD, 4; SEM, 1.2) (**Figure 1A**). The percentage of reactive H1975 cells in control samples were between min–max of 0.2%–4% (mean, 1; SD, 1.1; SEM, 0.3) versus chemotherapy- treated group reaching maximum 5.8% (min–max, 0.7%–5.8%; SD, 1.6; SEM, 0.5; *p<0.05). The ICI treatment led to the production of H1975 binding antibodies between min–max of 0%–32.2% (mean, 6.1; SD, 9.7; SEM, 3) (**Figure 1B**). The percentage of reactive H1650 cells in control samples were between min–max of 0.1%–2.5% (mean, 0.9; SD, 0.7; SEM, 0.2) versus chemotherapy- treated group reaching a maximum of 8.1% (min–max, 2.1%–8.1%; SD, 1.8; SEM, 0.5; *p<0.05). The ICI treatment led to the production of H1650 binding antibodies between min–max of 0.1%–6.2% (mean, 2.7; SD, 2.3; SEM, 0.7) (**Figure 1C**).

### 3.2 The pattern of immune-oncology mediators in the plasma of platinum-based chemotherapy and second- or multiple-line PD-1 blockade- treated patients

The multiplex Luminex MagPix technology was used to measure the concentration of 17 soluble immune checkpoint modulators in the plasma samples of platinum-based chemotherapy (cisplatin or carboplatin; Chem.) or second- or multiple-line PD-1 blockade immune checkpoint inhibitor (Nivolumab or Pembrolizumab; ICI)- treated patients versus non-smoker healthy controls (Contols) or versus smoker healthy controls, respectively. The list of the proteins measured in the plasma of the human subjects enrolled in the study including full name, alternative name, gene ID, Uniprot ID, and the range of the detection is summarized in **Supplementary Table S2**. The individual concentrations of the cytokine/chemokines/immune checkpoint modulators are demonstrated in scatter plots in **Supplementary Figure S4**. The following markers were detected in significantly higher concentration in the plasma of Chem. group versus healthy non-smoker and smoker controls: BTLA, CD27, CD28, CD40, CD80, CD86, GITRL, ICOS, LAG-3, PD-1, PD-L1, and TLR-2. The following markers were detected in significantly higher concentration in the plasma of ICI group versus healthy non-smoker and smoker controls: CD27, CD28, CD40, GITRL, LAG-3, PD-1, PD-L1, and TLR-2 (**Table 3**; **Supplementary Figure S4**). There was no marker showing significantly different concentrations between the non-smoker controls and smoker controls. The CD80 and ICOS were increased in the Chem. group but not in the ICI group in relation with the controls.

**Table 3:** The summary of the soluble immune checkpoint modulator plasma concentrations in healthy non-smoker controls (Controls, n=10), in healthy smoker controls (n=9), in Chem. (n=10) or in the ICI (n=10) groups (Cont., n=10).

Marker	Cohort	Mean (pg/ml)	SD	SEM	CI (95%)
					0
**BTLA**	**Cont.**	2,644	2,773	876.9	660.2 –4628
	**Smoker control**	2,622	1,384	461.2	1,559 –3,686
	**Chem.**	7,168	3,467	1,096	4,688 –9,649
	**ICI**	6,283	1,999	632.2	4,853 –7,713
**CD27**	**Cont.**	663.4	417	131.9	365.1 –961.7
	**Smoker control**	562.8	315.3	105.1	320.5 –805.2
	**Chem.**	1,416	428.9	135.6	1,109 –1,723
	**ICI**	1,307	404.5	127.9	1,018 –1,597
**CD28**	**Cont.**	6,308	6,304	1,993	1,799 –10,818
	**Smoker control**	6,224	3,957	1,319	3,182 –9,265
	**Chem.**	17,623	9,540	3,017	10,798 –24,447
	**ICI**	17,726	7,738	2,447	12,190 –23,261
**CD40**	**Cont.**	322.7	143.2	45.28	220.3 –425.2
	**Smoker control**	260.1	111.8	37.28	174.2 –346.1
	**Chem.**	633.3	155.7	49.22	522 –744.6
	**ICI**	615.6	146.5	46.32	510.8 –720.4
**CD80/B7-1**	**Cont.**	456.1	457.3	144.6	129 –783.2
	**Smoker control**	416.6	228.4	76.12	241 –592.1
	**Chem.**	1,063	455.8	144.1	737.1 –1,389
	**ICI**	955.7	286.6	90.63	750.7 –1,161
**CD86/B7-2**	**Cont.**	1,440	1,944	614.9	49.44 –2,831
	**Smoker control**	1,110	695.8	231.9	574.7 –1,644
	**Chem.**	4,920	3,020	954.9	2,760 –7,080
	**ICI**	4,412	2,159	682.7	2,868 –5,957
**CTLA-4**	**Cont.**	168	206.2	65.22	20.46 –315.5
	**Smoker control**	167.3	99.0	33.0	91.21 –243.7
	**Chem.**	469.3	243.9	77.11	294.9 –643.8
	**ICI**	425.7	163.5	51.69	308.8 –542.6
**GITR**	**Cont.**	366.4	508.9	160.9	2.37 –730.4
	**Smoker control**	405.1	274.3	91.44	194.3 –616.0
	**Chem.**	1,363	799.4	252.8	791.4 –1,935
	**ICI**	1,370	712.4	225.3	859.9 –1,879
**GITRL**	**Cont.**	1,798	1,439	455	768.9 –2,828
	**Smoker control**	1,582	807.3	269.1	961.2 –2,202
	**Chem.**	3,790	1,411	446.2	2,781 –4,800
	**ICI**	3,859	1,061	335.4	3,100 –4,617
**HVEM**	**Cont.**	538.3	193.7	61.24	399.7 –676.8
	**Smoker control**	374.7	181.6	60.54	235.1 –514.4
	**Chem.**	981.4	375.5	118.7	712.8 –1,250
	**ICI**	923.7	348	110.1	674.7 –1,173
**ICOS**	**Cont.**	2,911	3,449	1,091	444.3 –5,378
	**Smoker control**	2,913	1,834	611.4	1,503 –4,323
	**Chem.**	7,712	3,989	1,262	4,858 –10,566
	**ICI**	6,564	2,126	672.1	5,043 –8,084
**LAG-3**	**Cont.**	35,046	36,480	11,536	8,950 –61,142
	**Smoker control**	30,775	18,096	6,032	16,866 –44,685
	**Chem.**	84,508	33,650	10,641	60,437 –108,580
	**ICI**	88,290	21,631	6,840	72,816 –103,763
**PD-1**	**Cont.**	1,318	1,489	470.9	252.5 –2,383
	**Smoker control**	1,158	681.9	227.3	633.6 –1,682
	**Chem.**	3,766	1,906	602.6	2,403 –5,130
	**ICI**	4,763	1,383	437.4	3,773 –5,752
**PD-L1**	**Cont.**	322.1	398.8	126.1	36.78 –607.4
	**Smoker control**	323.9	197	65.65	172.5 –475.3
	**Chem.**	986.5	481.2	152.2	642.3 –1,331
	**ICI**	955.3	351	111	704.2 –1,206
**PD-L2**	**Cont.**	4,934	571.1	180.6	4,525 –5,342
	**Smoker control**	3,753	672.7	224.2	3,236 –4,270
	**Chem.**	5,588	867	274.2	4,967 –6,208
	**ICI**	5,407	896.8	283.6	4,766 –6,049
**TIM-3**	**Cont.**	974.3	332.4	105.1	736.5 –1,212
	**Smoker control**	645	183.9	61.31	503.7 –786.4
	**Chem.**	1,526	454.6	143.7	1,201 –1,852
	**ICI**	1,512	556	175.8	1,114 –1,910
**TLR-2**	**Cont.**	2,754	3,170	1,003	486.1 –5,022
	**Smoker control**	2,407	1,630	543.3	1,154 –3,660
	**Chem.**	8,641	4,289	1,356	5,573 – 11,710
	**ICI**	7,750	3,145	994.6	5,500 –10,000

The arithmetic means (Mean), standard deviation (SD), and the standard error of the mean (SEM) of the plasma marker concentrations were calculated. The multiple comparisons of the concentrations of each marker were carried out by the Kruskal–Wallis test. Significant differences are labeled in **Supplementary Figure S4**. The 95% confidence intervals (CI, 95%) were calculated for each marker in all groups, separately.

### 3.3 The effect of first-line platinum-based chemotherapy or multiple-line PD-1 blockade on the peripheral immunophenotype detected by single cell mass cytometry

**Figure 2:**
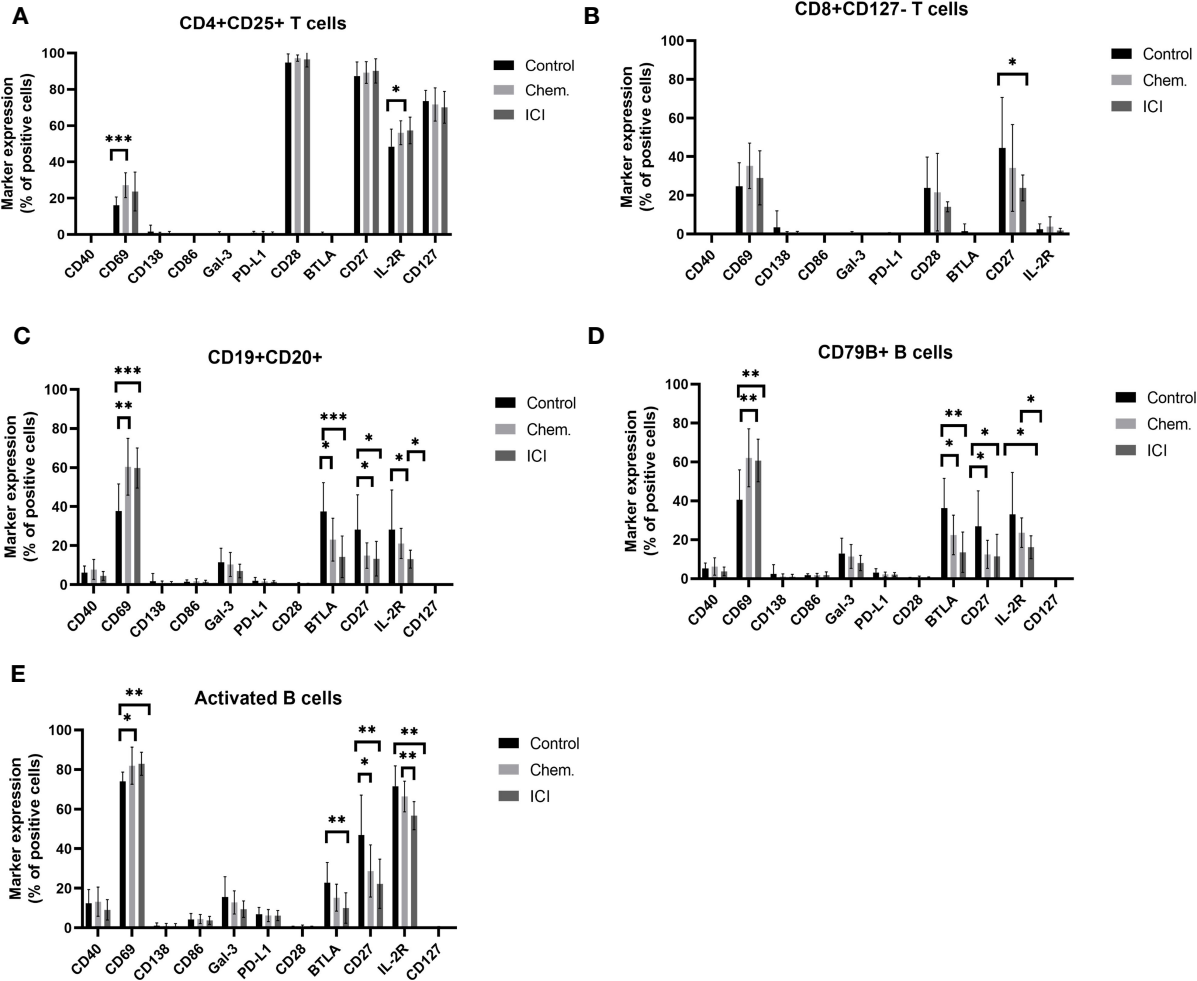
Expression profile of immune regulatory molecules on T- or B-cell subsets following platinum-based chemotherapy or PD-1 blockade. The PBMCs were purified from the peripheral blood of platinum-based chemotherapy (Chem.) or second- or multiple-line PD-1 blockade immunotherapy (ICI) patients and assayed for single- cell mass cytometry as described in *Materials and Methods.* The panel of the antibodies used for CyTOF is listed in **Table 2**. Manual gating was performed in Cytobank, and it is shown in **Supplementary Figure S5**. The T-cell **(A, B)** or B-cell subsets **(C–E)**, positive for the expression of cell surface immune regulatory molecules CD40, CD69, CD138, CD86, Gal-3, PD-L1, CD28, BTLA, CD27, IL-2R, and CD127, are shown as the percentages of parental population. *p < 0.05, **p < 0.01, ***p < 0.001.

Single- cell mass cytometry (CyTOF) was used for the immunophenotyping of patient-derived peripheral leukocytes. The expression of CD69 increased upon platinum-based chemotherapy 27.19% ± 6.81% versus 16.24% ± 4.47% (mean ± SD) of CD4+CD25+ T-cells. A slight increase in the percentage of IL-2R positivity was also observed upon chemotherapy (56.09% ± 6.58% vs. 48.42 ± 9.69%) (**Figure 2A**). Exhaustion of CD8+ T-cells was also measured by the decrease in the expression of costimulatory CD27 on the IL-7Rα chain (CD127 −) negative subpopulation in the average of the ICI patients compared to controls (23.9 ± 6.66 vs. 44.55 ± 26.09) (**Figure 2B**). Three B-cell populations were gated, the CD19+CD20+ B-cells, CD79B+ B-cells, and activated B-cells (CD19+CD25+/CD69+). The CD69^bright^ and IL-2R^bright^ positive cells were gated on B-cell variants. The CD69 is a type II C-type lectin involved in the migration of lymphocytes highly expressed upon activation in both T- and B-cells (31, 32). The CD69 bright cells increased in all gated B-cell subtypes upon chemotherapy and ICI compared to that in healthy controls (**Figures 2C–E**). The BTLA-negative regulator of activation was suppressed upon chemotherapy and ICI in CD19+CD20+ B-cells (**Supplementary Figure S6**) and CD79B+ B-cells, and only ICI led to the significant decrease in BTLA on activated B-cells (10.06 ± 7.72 vs. 22.72 ± 10.34 in controls) (**Figures 2C–E**). Representative viSNE plots show the downregulation of cell surface BTLA on Chem.- or ICI- treated samples on CD19+CD20+ B-cells (**Supplementary Figure S6**). The positive regulators of activation, both CD27 and IL-2R^bright^ populations, were significantly reduced upon chemotherapy or ICI in the average of the study cohorts on the surface of all investigated B-cell subtypes (**Figures 2C–E**).

**Figure 3:**
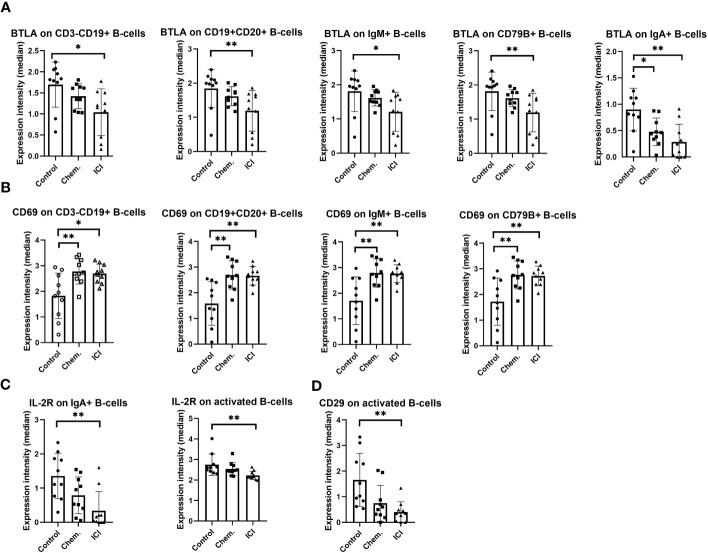
The expression intensity of BTLA, CD69, IL-2R, and CD29 on different B-cell subsets. The PBMCs were purified from the peripheral blood of platinum-based chemotherapy (Chem.) or multiple-line PD-1 blockade immunotherapy (ICI) patients and assayed for single- cell mass cytometry as described in *Materials and Methods*. The panel of the antibodies used for CyTOF is listed in **Table 2**. Manual gating was performed in Cytobank, and it is shown in **Supplementary Figure S5**. The median metal intensities for **(A)** BTLA, **(B)** CD69, **(C)** IL-2R, and **(D)** CD29 are proportional with marker densities on the surface of the analyzed B-cell subsets **(A–D)**. *p < 0.05, **p < 0.01.

Following the frequencies of T- or B-cell subsets, marker expression intensities (arcsinh-transformed medians) were investigated. The negative regulator of immune activation BTLA was significantly decreased in CD3-CD19+ and CD19+CD20+ conventional B-cells, and in IgM+ B-cells, CD79B+, and in IgA+ B-cells (**Figure 3A**). In parallel, the CD69 involved in lymphocyte activation and migration was increased in CD3-CD19+ and CD19+CD20+ conventional B-cells, IgM+ B-cells, and in the B-cell receptor component CD79B+ B-cells (**Figure 3B**). However, for markers important for activation or extravasation, the IL-2R (CD25) was decreased in the cell surface of IgA+ B-cells and activated B-cells (**Figure 3C**), and similarly, the CD29 (beta 1 integrin) was also decreased in activated B-cells (**Figure 3D**).

**Figure 4:**
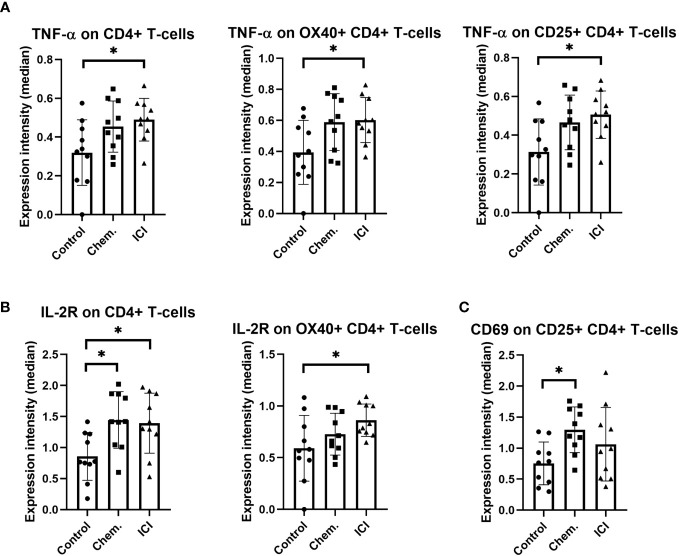
The expression intensities of TNF-α, IL-2R, and CD69 on different CD4+ T-cell subsets. The PBMCs were purified from the peripheral blood of platinum-based chemotherapy (Chem.) or multiple-line PD-1 blockade immunotherapy (ICI) patients and assayed for single- cell mass cytometry as described in *Materials and Methods*. The panel of the antibodies used for CyTOF is listed in **Table 2**. Manual gating was performed in Cytobank, and it is shown in **Supplementary Figure S5**. The median metal intensities for **(A)** TNF-α, **(B)** IL-2R, and **(C)** CD69 are proportional with marker expression intensities of the analyzed CD4+ T-cell subsets **(A–C)**. *p < 0.05.

The CD4+ helper T-cells may support both the humoral and cellular arms of the immune defense against the malignant cells. The second- line anti-PD-1 blocking ICI therapy increased the TNF-α in CD4+ T-cells and in costimulatory OX40+/CD4+ or CD25+/CD4+ T-cell populations (**Figure 4A**). The IL-2R density also increased upon multiple-line ICI treatment on CD4+ T-cells or on CD4+/OX40+ T-cells (**Figure 4B**). The platinum-based chemotherapy increased CD69 expression on CD4+/CD25+ T-cells (**Figure 4C**).

## 4 Discussion

The manifestation of most type of solid tumors occurs following the escape from immunosurveillance (33). A clinical study with 931 patients recruited between 2013 and 2020 identified a significant correlation between smoking shistory and higher tumor mutational burden in non-small cell lung cancer, lung adenocarcinoma (34). Additionally, in the case of lung cancer, the tobacco- smoking-generated airway inflammation further skews the polarization of immune cells to a tumor promoter phenotype (3, 21, 35). Previously, we have shown the immunomodulatory effect of cisplatin in triple negative murine breast cancer model reducing the emergence of splenic CD44+, IL-17A+ myeloid suppressor cells (25). Here, we focused on smoker human NSCLC, adenocarcinoma patients receiving platinum-based chemotherapy, or multiple-line PD-1 blocking immunotherapy. In our cross-sectional study, cisplatin or carboplatin were used as first-line platinum-based chemotherapeutics (Chem. group), or Nivolumab and Pembrolizumab were used for PD-1 blocking second- or multiple-line immunotherapy (ICI group) enrolled between November 2018 and June 2019. The OS of the ICI- treated patients was significantly improved (**Supplementary Figure S1**). We have developed a flow-cytometry-based assay to measure the humoral immunity against NSCLC-derived antigens. The human NSCLC cell lines (A549, H1975, and H1650) were incubated with NSCLC-patient-derived plasma and assayed for IgG antibodies bound on the surface of the NSCLC cell lines bearing putative NSCLC-related cell surface antigens. It has to be emphasized that using three different human cell lines instead of the corresponding patient- matched self-reactive tumor biopsy specimens to detect tumor reactive IgG level is a limitation of our study, but the access to patient-matched lung cancer fresh biopsy was not available. However, using the A549, H1975, and H1650 recipient cells, we were able to show a significant increase in NSCLC- reactive IgG antibodies in plasma upon chemotherapy and a tendentious increase upon multiple-line ICI therapy reaching up to 32% positivity of H1975 cells in one well-responder case. Further analysis of NSCLC tumor cell line reactive plasma samples of well- responder cases may help to identify IgG sequences for the development of therapeutic antibodies, but this work is beyond the capacities of our laboratory. Another important component of patient-derived plasma samples, soluble immune checkpoint mediators were quantitatively measured using the multiplex Luminex MagPix system. The lung cancer patients recruited for our cross-sectional study were in advanced stage receiving chemotherapy or second-line ICI following failed chemotherapy. Authors may suppose that this was the reason why the Chem. and ICI groups were not differentiated in terms of the plasma concentrations of the 17 soluble markers. However, the following markers were detected in significantly higher concentration of the plasma of Chem. group versus healthy non-smoker and smoker controls: BTLA, CD27, CD28, CD40, CD80, CD86, GITRL, ICOS, LAG-3, PD-1, PD-L1, and TLR-2. The following markers were detected in significantly higher concentration in the plasma of ICI group versus healthy non-smoker and smoker controls: CD27, CD28, CD40, GITRL, LAG-3, PD-1, PD-L1, and TLR-2. The increased concentration of CD80 and ICOS was present only in the Chem. group differentiating from the ICI group in relation with the controls. There was no marker showing significantly different concentration between the non-smoker controls and smoker controls. The functional categories of these soluble mediators represent immune checkpoint inhibitors such as BTLA, LAG-3, PD-1. PD-L1, and PD-L2; decoy TLR2 inhibiting innate activation upon danger signals; or costimulatory molecules such as CD27, CD28, CD40, CD80, CD86, GITRL, and ICOS (36–39). This mixed phenotype of the elevated inhibitory and costimulatory molecules may be associated with cancer-driven inflammation and may counteract with the success of ICI therapy. Next, we aimed to deeply analyze the immunophenotype of the peripheral immune system, comparing the PBMCs in Chem. group with multiple-line ICI- treated NSCLC patients using the state-of-the-art single cell mass cytometry (CyTOF). A mixed immunophenotype was detected, both the immunostimulatory effect of platinum-based chemotherapeutics and the exhaustion of CD4+ or CD8+ T-cells were shown. The BTLA was identified as a negative regulator of humoral immune activation inhibiting the IL-6 pathway (40, 41). Our CyTOF experiments showed downregulation of BTLA on conventional, CD19+; CD19+/CD20+ B-cells, IgM+; IgA+, or CD79B+ B-cells following the PD-1 blocking therapy. The increased expression of TNF-α or IL-2R in CD4+; CD4+/OX40+ T-cells showed the immunoactivation. However, one obstacle of cancer immunotherapy is the exhaustion of T-cells, which was shown by the decrease in CD27 and CD127 on CD8+ T-cells upon ICI therapy in our current study. Moreover, the IL-2R and CD29 were decreased on IgA+ or activated B-cells. The role of CD69 is controversial because it is a type II transmembrane glycoprotein with a C-type lectin domain that is a marker of early lymphocyte activation (42). However, deficiency in CD69 in animal models or targeting CD69 showed an attenuated tumor growth and improved anti-tumor immunity (43, 44). Therefore, CD69 is becoming a factor regulating anti-tumor immunity through T-cell exhaustion (45). In line with this, a study conducted on the Cancer Treatment Response gene signature DataBase revealed CD69 expression as a prognostic factor for responding to PD-1 blocking therapy (46). In our experiments, CD69 was upregulated in the percentages of CD4+CD25+ helper T-cells, conventional CD19+CD20+ B-cells, and in the frequency of CD79B+ B-cells, or activated B-Cells (CD19+, CD69^bright^ IL-2R^bright^). Analyzing the median expression intensities, the cell surface density of CD69 was also increased on B-cell subsets: on CD3-CD19+ and CD19+CD20+ conventional B-cells, IgM+ B-cells, and on the B-cell receptor component CD79B+ B-cells. B-cell activation may result in the production of tumor- specific antibodies, and B-cells can present antigens to CD4 or CD8 T-cells facilitating cellular immunity also (47). Higher CD69 expression on CD19+ B-cells was reported with longer survival in colorectal cancer (48).Understanding the complexity of the polarization of T- or B-cell subsets, myeloid cell types in response to ICI therapy is the focus of the current research. In agreement with our study, others reported also the “Janus” scenario regarding the anti-tumor immune response following ICI therapy. Sorin et al. published the spatial single- cell immunophenotyping of the tumor microenvironment of 27 NSCLC patients following ICI and found that CXCL13 expression on CD4+ T-cells was associated with good prognosis (49). Rahim et al. showed in head and neck squamous cell carcinoma that exhausted CD8+ T-cells reduced in frequency following ICI but localized nearer to DCs in the lymph nodes (50). Luo et al. identified CD103+ CD39+ T-cells in colorectal cancer patients with an exhausted but cytotoxic phenotype as a good prognosis in response to ICI therapy (51). Lavoie et al. showed that PD1 − CD4+ T cells had higher TNFα and higher CCR4 expression, while their PD1+ CD4+ T cells had higher interferon-γ and lower CCR4 expression in non-responder cases to ICI in urothelial and renal cell carcinoma (52). Xiao et al. analyzed 26 melanoma patients who underwent PD-1 blocking therapy and showed the abundance of CD27+ and TIM-3+ T-cells in the tumor microenvironment of well- responders. Sidiropoulos et al. analyzed the T-cell polarization states in pancreatic ductal adenocarcinoma, melanoma, and hepatocellular carcinoma upon immunotherapies and established single-cell trajectory inference and non-negative matrix factorization methods to CyTOF data to trace the dynamics of T- cell states (53). Their state-of-the art method is demonstrated to monitor patient-specific T-cell states including naive, memory, and effector T- cell phenotypes during immunotherapy.

Taken together, we could show the humoral immune activation in the Chem. group, since the anti-tumor IgG antibodies were significantly increased following chemotherapy (**Figure 1**). In line with this, CD69 and CD79 were also upregulated after chemotherapy on the cell surface of B-cells (**Figure 2**). The ICI therapy enhanced the effect of chemotherapy in the increase in IL-2R on CD4+CD25+ T-cells and increased the decline of CD27 on CD8+CD127 − T-cells (**Figure 2**). The ICI therapy potentiated the effect of chemotherapy in the decline of BTLA on B-cell subsets and in the increase in CD69 on B-cell subsets (**Figure 3**). Looking at **Figure 4**, it is also turned out that chemotherapy induced TNF-α in CD4+ T-cells, in CD4+OX40+ T-cells, and in CD4+CD25+ T-cells and that TNF-α induction was further increased by second-line ICI therapy. However, only ICI provided statistically significant TNF-a induction.

Finally, limitations of our study should be mentioned: (1) cell lines were used as a model system expressing putative NSCLC -related antigens, (2) second- or multiple-line application of PD-1 blockade therapy, (3) cross-sectional study without longitudinal follow-up of the soluble mediators or cell surface markers, (4) relatively small number of subjects in the study cohorts, and (5) the complex immunophenotyping of smoker but non-tumorous cases would be relevant, but in those cases, the appearance and disappearance of previous tumors could not be ruled out. Therefore, the authors suggest that the analysis of smoking on the immunophenotype without the manifestation of solid tumors should be a proposed future study. However, we could show the appearance of NSCLC tumor cell line reactive IgG antibodies in the plasma of the patients. The concentration of 17 soluble mediators, immune checkpoint regulators, were measured in advanced NSCLC patients. Moreover, single- cell mass cytometry showed a Janus-faced immunophenotype with the emergence of immune activation in line with T-cell exhaustion. Further research is warranted about the complex immunophenotype on first-line PD-1 blockade with prospective follow-up in comparison with platinum-based chemotherapy.

## Data availability statement

The original contributions presented in the study are included in the article/**Supplementary Material**. Further inquiries can be directed to the corresponding authors.

## Ethics statement

The studies involving humans were approved by Ethics Committee of the National Public Health Center, Hungary under the 33815-7/2018/EÜIG Project identification code and by the Ethics Committee of the University of Szeged under the 163/2018-SZTE Project identification code. The studies were conducted in accordance with the local legislation and institutional requirements. The participants provided their written informed consent to participate in this study.

## Author contributions

GL, LP, and GS conceived the project. GS and KS was involved in the experimental design. PN, NG, JB, LT, and JF performed the experiments. NG, PN, and GS analyzed the data. GS drafted the manuscript. All authors contributed to the article and approved the submitted version.
