# Supplementary material for: Single-cell mass cytometric analysis of peripheral immunity and multiplex plasma marker profiling of non-small cell lung cancer patients receiving PD-1 targeting immune checkpoint inhibitors in comparison with platinum-based chemotherapy
Source: Front Immunol. 2023 Oct 13;14:1243233. doi: 10.3389/fimmu.2023.1243233 (PMC10611454; doi:10.3389/fimmu.2023.1243233)
Supplement: Supplementary file 1 [file DataSheet_1.docx]

Supplementary Material

Single-Cell Mass Cytometric Analysis of Peripheral Immunity and Multiplex Plasma Marker Profiling of Non-Small Cell Lung Cancer Patients Receiving PD-1 Targeting Immune Checkpoint Inhibitors in Comparison with Platinum-Based Chemotherapy

Patrícia Neuperger^1,2^, Klára Szalontai^3^, Nikolett Gémes^1,2^, József Á. Balog^1^, László Tiszlavicz^4^, József Furák^5^, György Lázár^5^, László G. Puskás^1,6,*^, Gábor J. Szebeni^1,7,8,*^

*** Correspondence:** These authors contributed equally to this work. Laszlo G. Puskas, laszlo@avidinbiotech.com, Gabor J. Szebeni, szebeni.gabor@brc.hu, Tel.: +3662599782

**Supplementary Table 1.** Clinical data of the enrolled patients including histology type, PFS, OS, therapy, T-N-M stage and comorbidities. *See the attached Excel sheet.*

**Supplementary Table 2.** The list of the proteins measured in the plasma of the human subjects enrolled in the study. *See the attached pdf.*

**
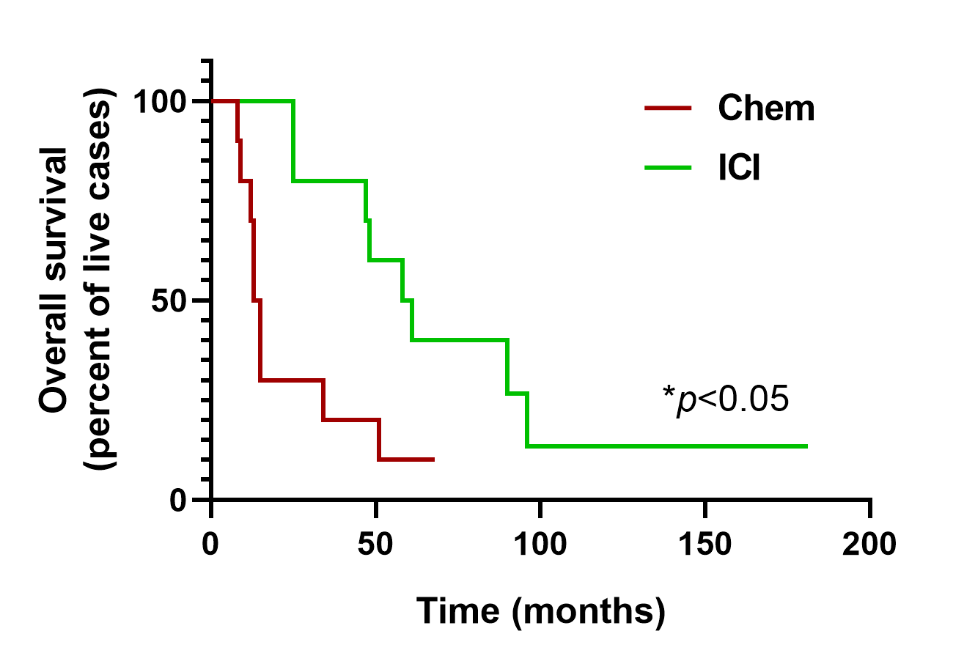
**

**Supplementary Figure 1.** Overall survival of the Chem or ICI treated groups. The log-rank test showed significant improvement of the overall survival in the ICI treated group. *p<0.05


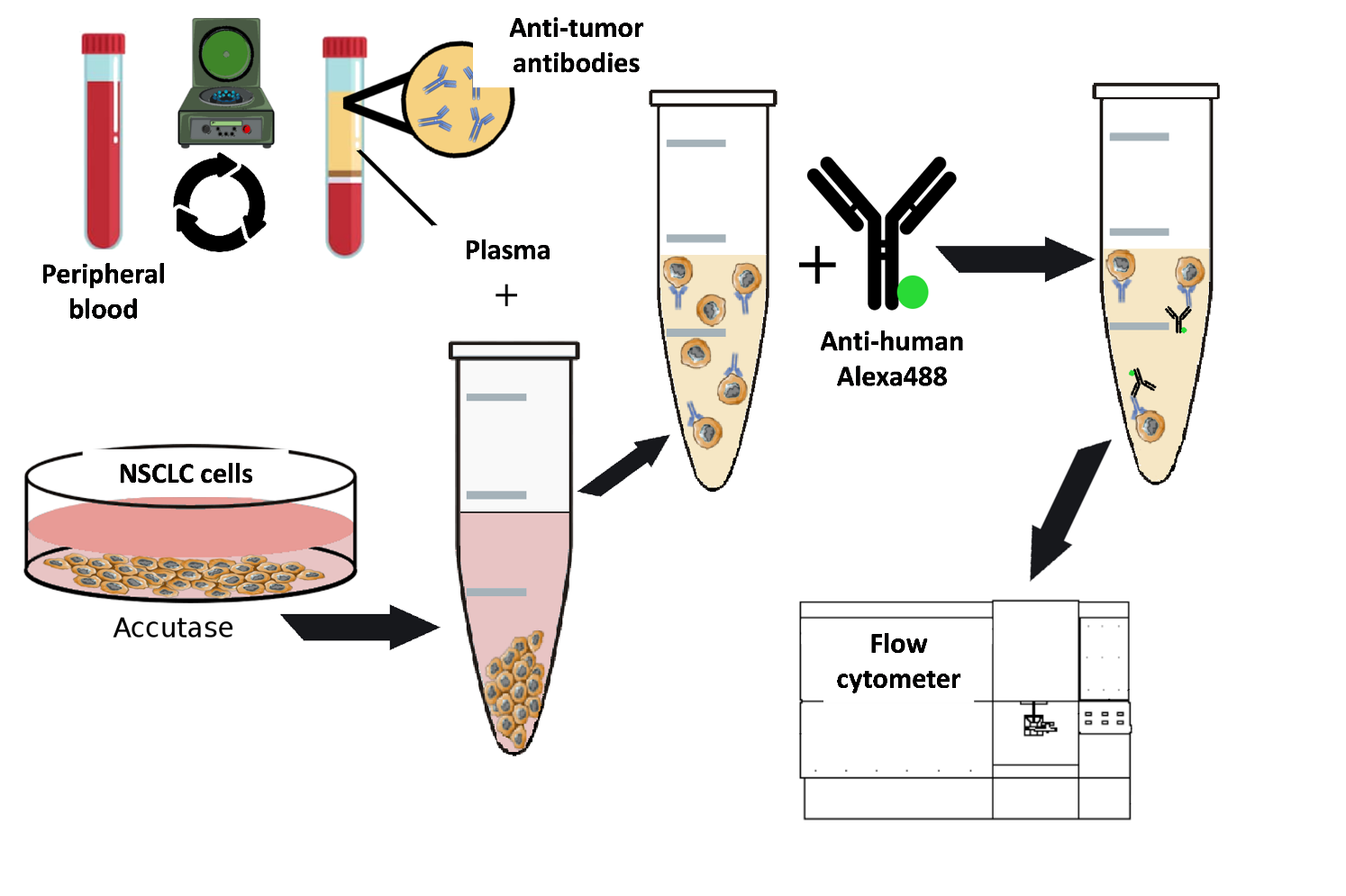


**Supplementary Figure 2.** The schematic cartoon of the assay detecting NSCLC cell line cell surface antigen recognizing IgG antibodies of patient-derived plasma samples. Detailed explanation is in the Materials and Methods section.

**
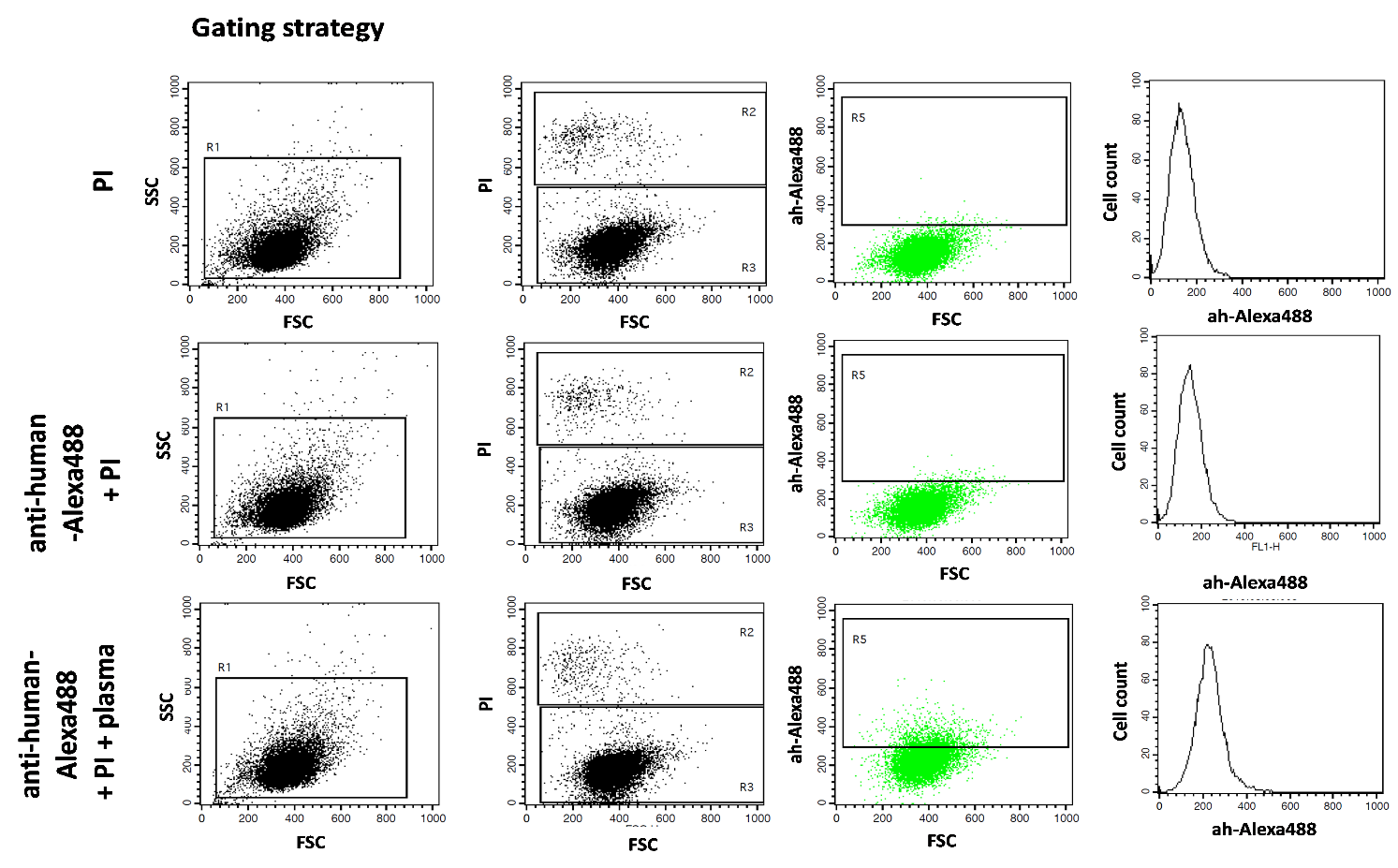
**

**Supplementary Figure 3.** Representative flow-cytometry gating on NSCLC cell lines (R1) incubating with patient-derived plasma. Propidium iodide was used to gate on viable cells (R2), and subsequently gating was on anti-humanAlexa488 reactive cells (R3). Histograms shows the increased fluorescence intensity in one representative example of NSCLC reactive IgG containing patient-derived plasma.

**
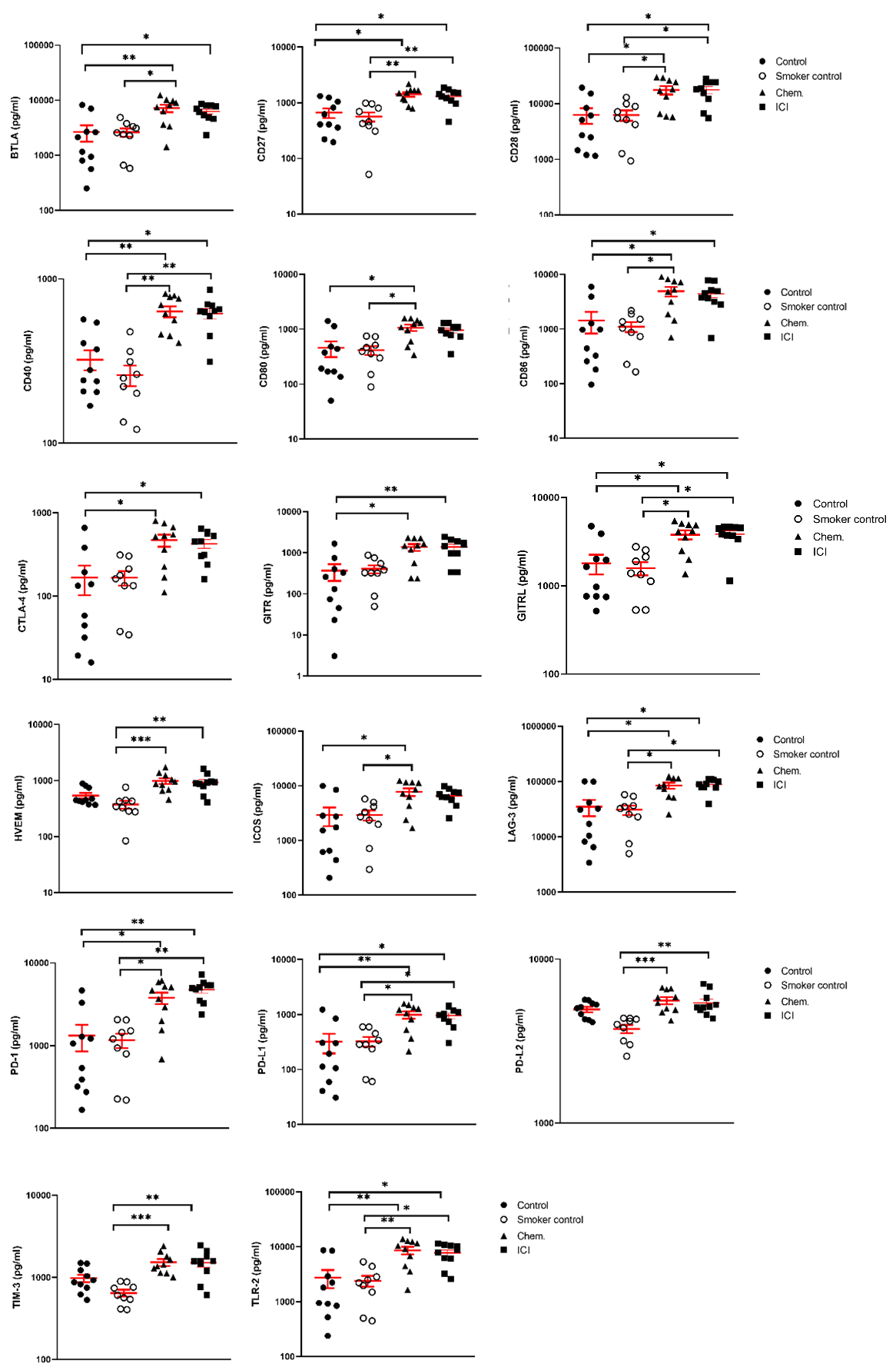
**

**Supplementary Figure 4.** Scatter plots show the individual values of the measured soluble markers in healthy controls, smoker controls, platinum-based chemotherapy treated (Chem.) or multiple-line ICI treated samples assayed by the Luminex MagPix system.

**
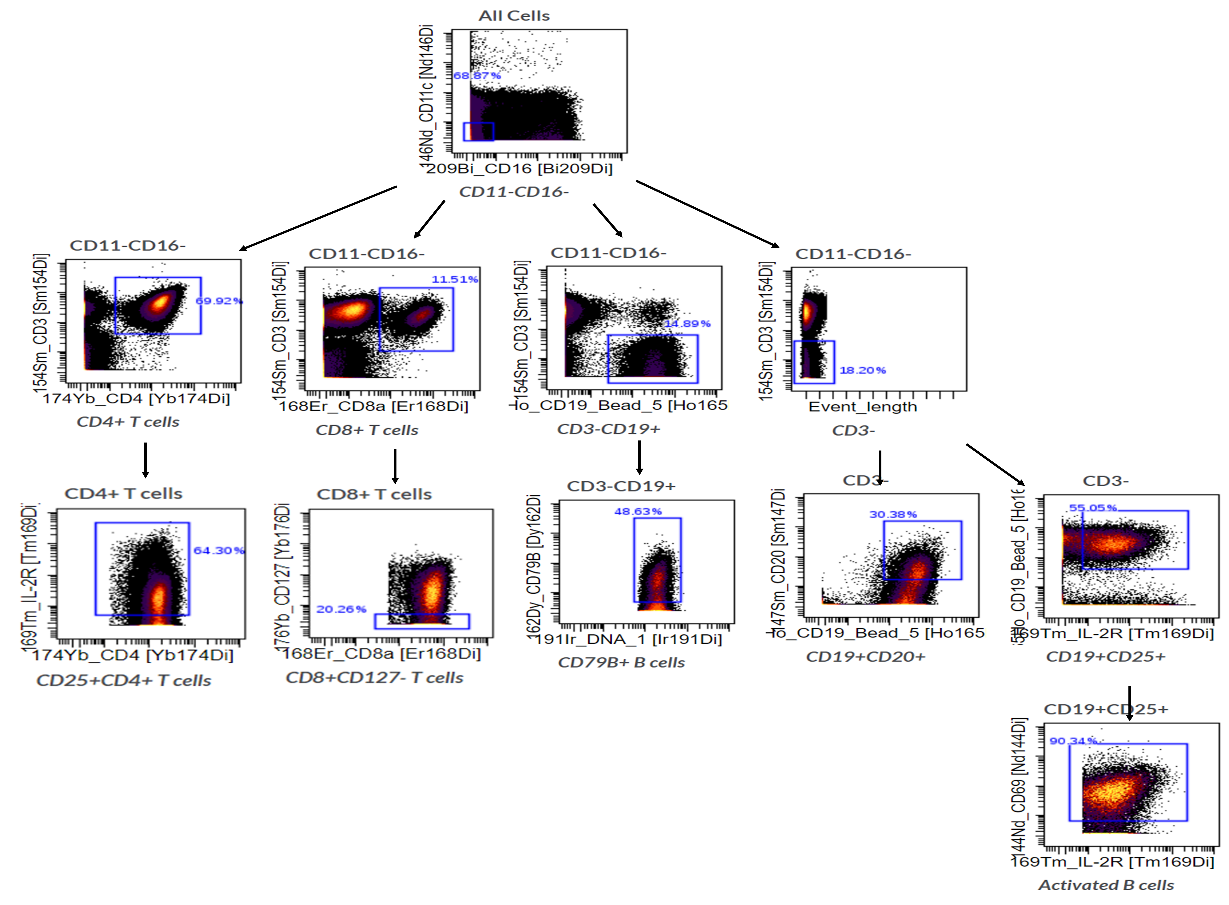
**

**Supplementary Figure 5.** The manual gating strategy is shown to define CD4+CD25+ T-cells, CD8+CD127- T-cells, CD19+CD20+ B-cells, CD79B+ B-cells, or activated B-cells (CD19+CD25+CD69+). The manual gating was performed in Cytobank (Beckman Coulter). Only representative plots of populations are shown where significant differences were detected among the study cohorts.

**
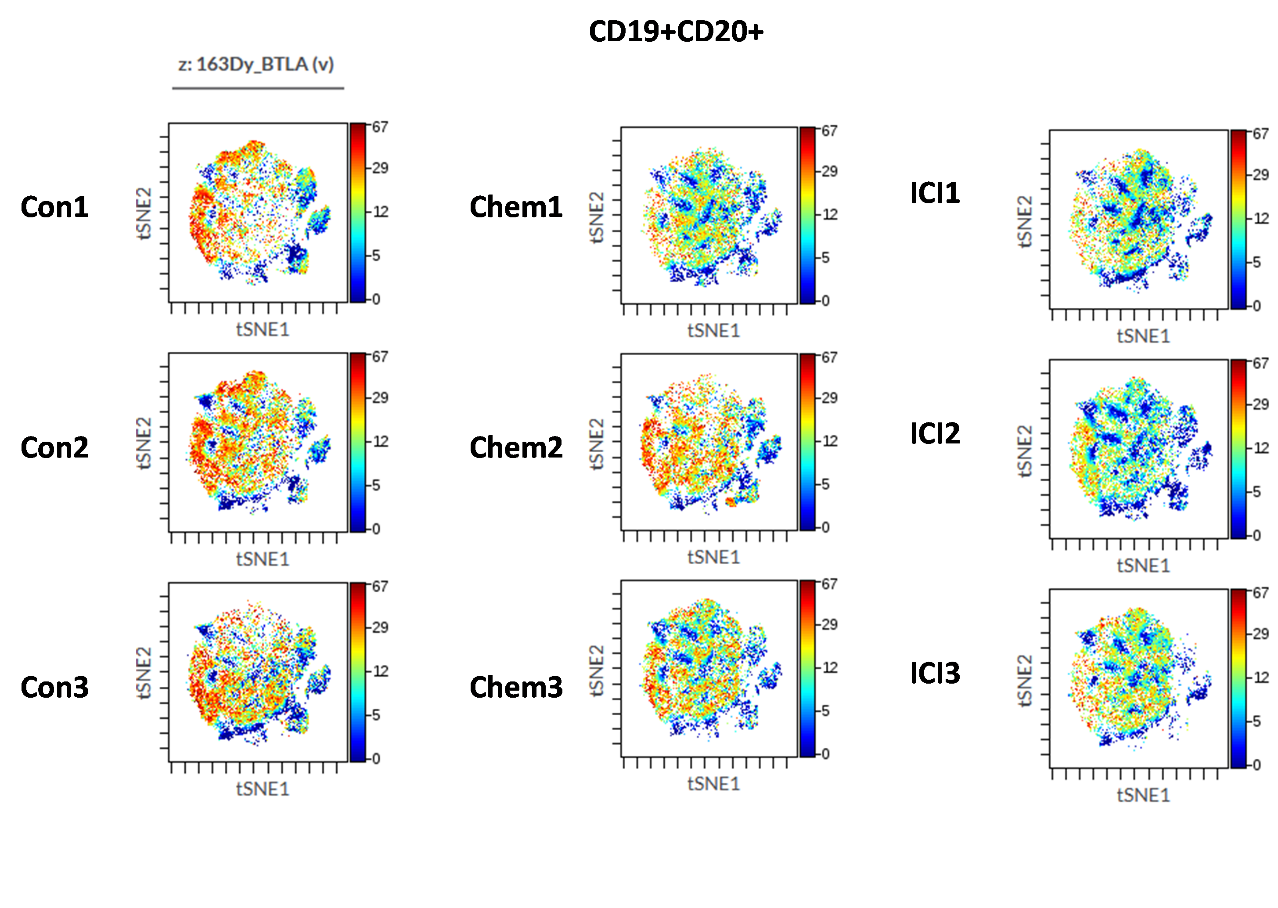
**

**Supplementary Figure 6.** Representative viSNE plots (visualization of stochastic neighbor embedding) showing the downregulation of cell surface BTLA expression on CD19+CD20+ B-cells.
